# Involvement of β‐adrenoceptors in the cardiovascular responses induced by selective adenosine A_2A_ and A_2B_ receptor agonists

**DOI:** 10.1002/prp2.975

**Published:** 2022-05-29

**Authors:** Edward S. Wragg, Patrizia Pannucci, Stephen J. Hill, Jeanette Woolard, Samantha L. Cooper

**Affiliations:** ^1^ 6123 Division of Physiology, Pharmacology and Neuroscience School of Life Sciences University of Nottingham Nottingham UK; ^2^ Centre of Membrane Proteins and Receptors University of Birmingham and University of Nottingham Midlands UK

**Keywords:** A_2A_ receptor, A_2B_ receptor, adenosine, hemodynamics, β‐adrenoceptor

## Abstract

A_2A_ and A_2B_ adenosine receptors produce regionally selective regulation of vascular tone and elicit differing effects on mean arterial pressure (MAP), whilst inducing tachycardia. The tachycardia induced by the stimulation of A_2A_ or A_2B_ receptors has been suggested to be mediated by a reflex increase in sympathetic activity. Here, we have investigated the role of β_1_‐ and β_2_‐adrenoceptors in mediating the different cardiovascular responses to selective A_2A_ and A_2B_ receptor stimulation. Hemodynamic variables were measured in conscious male Sprague‐Dawley rats (350–450 g) via pulsed Doppler flowmetry. The effect of intravenous infusion (3 min per dose) of the A_2A_‐selective agonist CGS 21680 (0.1, 0.3, 1.0 µg.kg^−1^.min^−1^) or the A_2B_‐selective agonist BAY 60–6583 (4.0, 13.3, 40.0 µg.kg^−1^.min^−1^) in the absence or following pre‐treatment with the non‐selective β‐antagonist propranolol (1.0 mg.kg^−1^), the selective β_1_‐antagonist CGP 20712A (200 µg.kg^−1^), or the selective β_2_‐antagonist ICI 118,551 (2.0 mg.kg^−1^) was investigated (maintenance doses also administered). CGP 20712A and propranolol significantly reduced the tachycardic response to CGS 21680, with no change in the effect on MAP. ICI 118,551 increased BAY 60–6583‐mediated renal and mesenteric flows, but did not affect the heart rate response. CGP 20712A attenuated the BAY 60–6583‐induced tachycardia. These data imply a direct stimulation of the sympathetic activity via cardiac β_1_‐adrenoceptors as a mechanism for the A_2A_‐ and A_2B_‐induced tachycardia. However, the regionally selective effects of A_2B_ agonists on vascular conductance were independent of sympathetic activity and may be exploitable for the treatment of acute kidney injury and mesenteric ischemia.

## Graphical abstract

Involvement of β‐adrenoceptors in the cardiovascular responses induced by adenosine A_2A_ and A_2B_ receptor agonists.

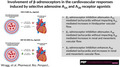

**Abbreviations**
A_1_R: Adenosine A_1_ receptor
A_2A_R: Adenosine A_2A_ receptor
A_2B_R: Adenosine A_2B_ receptor
A_3_R: adenosine A_3_ receptor
BRET: bioluminescence resonance energy transfer
cAMP: cyclic adenosine monophosphate
GPCR: G protein‐coupled receptor
HR: heart rate
HVC: hindquarters vascular conductance
MAP: mean arterial pressure
MVC: mesenteric vascular conductance
NTS: nucleus of the solitary tract
RVC: renal vascular conductance
VC: vascular conductance

## 1 INTRODUCTION

Adenosine is a purine nucleoside that has an important role within the cardiovascular system.^1^, ^2^ The physiological actions of adenosine are the consequence of its interaction with four different G protein‐coupled receptors (GPCRs), namely the adenosine A _1_ , A _2A_ , A _2B_ , and A _3_ receptors (A_1_R, A_2A_R, A_2B_R, A_3_R).^3^, ^4^ By interacting with these GPCRs, adenosine and its analogues initiate defined signaling pathways that provoke different biological effects on numerous organ systems.^5^, ^6^ A_1_ and A_3_ adenosine receptors primarily couple to the inhibitory G‐proteins G_o_ or G_i_ suppressing cyclic adenosine monophosphate (cAMP) production, while, in contrast, A_2A_ and A_2B_ subtypes preferentially activate stimulatory G_s_ proteins, thus increasing intracellular cAMP concentrations.^4^, ^7^ A_2A_ and A_2B_ receptors are widely expressed in the cardiovascular system, where their regulation plays a key modulatory role in controlling heart rate and blood pressure.^8^, ^9^, ^10^ In addition to regulating heart rate (HR), cardiac contraction, inflammation, and vascular remodeling,^3^, ^11^ both A_2A_ and A_2B_ receptors mediate systemic and pulmonary vasodilation.^12^, ^13^ Activation of these adenosine receptors in response to hypoxic or ischemic stress also plays an important role in the prevention of renal failure by promoting renal perfusion.^7^, ^14^ A_2A_ and A_2B_ receptors are therefore promising targets for a wide range of cardiovascular diseases, in particular hypertension and acute kidney injury.^7^, ^14^, ^15^

An in vivo evaluation of the cardiovascular effects of selective A_2A_ and A_2B_ agonists, CGS 21680 and BAY 60–6583, respectively, showed that A_2A_ and A_2B_ receptors exert a regionally selective control of vascular conductance.^10^ The A_2A_R subtype mediates vasodilatory effects in the hindquarters vascular bed, with minimal impact on the mesenteric and renal vasculature, whereas A_2B_Rs have been demonstrated to have major control of renal and mesenteric vascular tone but have no effect on hindquarters vascular conductance.^10^ In addition, the activation of both A_2_ receptor subtypes resulted in a parallel increase in HR, which may be secondary to a reflex response to the vasodilatation induced in different vascular beds.^10^

The arterial baroreflex is a neural mechanism that plays a crucial role in the fine regulation of blood pressure.^16^ The continuous sensation of blood pressure by tonic arterial baroreceptors allows this reflex to make constant adjustments of blood pressure by inducing rapid changes in heart rate and peripheral vascular resistance.^17^ Within the context of the neural control of cardiovascular function, the nucleus of the solitary tract (NTS) represents the first synaptic station for the processing of cardiovascular afferent inputs.^18^ In this regard, there is evidence of A_2A_ receptor involvement in the control of baroreflex activity via NTS,^19^ as well as A_2B_ receptor involvement in cardiovascular regulation via the posterior hypothalamus.^20^

Sympathetic activity and its effect on the cardiovascular system are crucial aspects of the baroreceptor reflex.^16^ Indeed, the modulation of the sympathetic outflow is under the control of baroreceptor afferent activity, which evokes changes in the sympathetic activity to maintain an adequate blood pressure.^21^ The physiological responses to sympathetic activation result from the interaction between catecholamines and adrenoceptors.^22^ In particular, β _1_ and β _2_ adrenoceptors play fundamental roles in the regulation of cardiovascular homeostasis.^23^, ^24^ To evaluate the contribution of β adrenoceptors to the tachycardia induced by selective A_2A_ and A_2B_ receptor stimulation, we have used the non‐selective β antagonist (propranolol), a β_1_ antagonist (CGP 20712A) and a β_2_ antagonist (ICI 118,551) to evaluate the extent to which the chronotropic and vasodilatory effects induced by selective A_2A_ (CGS 21680) and A_2B_ (BAY 60–6583) agonists result from an increase in sympathetic activity.

## 2 MATERIALS AND METHODS

### 2.1 Drugs, chemical reagents, and other material

4‐[2‐[[6‐Amino‐9‐(*N*‐ethyl‐β‐D‐ribofuranuronamidosyl)‐9*H*‐purin‐2‐yl]amino]ethyl]benzenepropanoic acid hydrochloride (CGS 21680 hydrochloride) (Cat#1063), 2‐[[6‐Amino‐3,5‐dicyano‐4‐[4‐(cyclopropylmethoxy)phenyl]‐2‐pyridinyl]thio]‐acetamide (BAY 60–6583) (Cat#4472), (*RS*)‐1‐[(1‐Methylethyl)amino]‐3‐(1‐naphthalenyloxy)‐2‐propanol hydrochloride (propranolol hydrochloride) (Cat#0624), 1‐[2‐((3‐Carbamoyl‐4‐hydroxy)phenoxy)ethylamino]‐3‐[4‐(1‐methyl‐4‐trifluoromethyl‐2‐imidazolyl)phenoxy]‐2‐propanol dihydrochloride (CGP 20712A dihydrochloride) (Cat#1024) and (±)‐*erythro*‐(S*,S*)‐1‐[2,3‐(Dihydro‐7‐methyl‐1*H*‐inden‐4‐yl)oxy]‐3‐[(1‐methylethyl)amino]‐2‐butanol hydrochloride (ICI 118,551 hydrochloride) (Cat#0821) were purchased from Tocris Bioscience (Bristol, UK). The fluorescent ligand, CA200645, was purchased from Hello Bio, (Bristol, UK) (Cat#HB7812). Dimethyl Sulfoxide (DMSO) (Cat#D5879) and Bovine Serum Albumin (BSA) (Cat#A7030) were purchased from Sigma‐Aldrich (Gillingham, UK). Furimazine (Cat#N1130) was purchased from Promega (Madison, WI, USA).

Fentanyl citrate was acquired from Martindale Pharmaceuticals (Essex, UK). Buprenorphine (Buprecare), Medetomidine Hydrochloride (Sedastart) and Atipamezole Hydrochloride (Sedastop) were obtained from Animalcare Ltd. (York, UK). Meloxicam (Metacam) was acquired from Boehringer Ingelheim Animal Health (Berkshire, UK). Heparin Sodium was obtained from Wockhardt (Wrexham, UK). Pentobarbitone (Euthatal) was obtained from Alstoe Animal Health (York, UK). Tween 80 (Cat#P1754) and propylene glycol (Cat#P4347) were purchased from Sigma‐Aldrich (Gillingham, UK).

### 2.2 Animals and surgery

Male Sprague‐Dawley rats (Charles River Laboratories, UK; 350–450 g) were used to perform these experiments. Animals were housed in pairs in a temperature‐controlled room (21–23°C) with a 12 h light‐dark cycle (lights on at 06:00) with free access to food (18% Protein Rodent Diet; Envigo, Madison WI, USA) and water. Upon arrival within the Unit, animals were housed during an acclimatization period of at least of 7 days prior to any surgery. All procedures were performed with approval from the University of Nottingham Animal Welfare and Ethical Review Board and performed in line with the Animals (Scientific Procedures) Act (1986), under UK Home Office approved Project License and Personal License authority. 53 rats were used during this study, and all animal experiments are reported in compliance with the ARRIVE guidelines^25^ and the editorial on reporting animal studies.^26^ Surgical procedures were carried out under general anesthesia (fentanyl and medetomidine, 300 µg.kg^−1^ each, i.p., supplemented as required). During the first surgery, miniature pulsed Doppler flow probes were implanted around the left renal and superior mesenteric arteries and the descending abdominal aorta to monitor haemodynamics.^27^ The probe wires were led subcutaneously to the nape of the neck, where they were taped and secured. Atipamezole hydrochloride (1 mg.kg^−1^, s.c.) and buprenorphine (30 µg.kg^−1^, s.c.) were provided as reversal agents and postoperative analgesia. A second dose of analgesia (buprenorphine 15 µg.kg^−1^, s.c.) was given 4 h post‐surgery. Supplementary analgesia (Meloxicam, 1 mg.kg^−1^.day^−1^, s.c) was administered for 3 days after surgery.

A second surgery was carried out at least 10 days after the surgical implantation of the vascular probes and after a satisfactory welfare inspection from the Named Veterinary Surgeon. During this surgery, performed under anesthesia (fentanyl and medetomidine, 300 µg.kg^−1^ each, i.p., supplemented as required), a catheter was implanted into the distal abdominal aorta via the caudal artery (to measure arterial blood pressure and heart rate), and three catheters were implanted into the right jugular vein (for drug administration).^27^ All catheters were led subcutaneously to the nape of the neck.

The probe wires were released from the nape of the neck to be soldered into a miniature plug (Omnetic connector corporation, USA), which was then mounted onto a custom‐designed harness worn by the rat. The catheters and probe wires were protected by a spring secured to the harness and attached to a counterbalanced pivot system to allow the free movement of the animal. Reversal of anesthetic and analgesia was administered (as described above). The arterial catheter was filled and infused with heparinized (15 U.ml^−1^) saline overnight to maintain potency.

Experiments began 24 h after surgery for catheter implantation, with animals fully conscious and unrestrained in home cages, with free access to food and water.

### 2.3 Cardiovascular recordings

During the cardiovascular monitoring periods, rats were connected to the customized data‐acquisition software (see below) via a tether system. Recordings were made for at least 30 min prior to the administration of any interventions and continuously for a minimum of 4 h thereafter. HR, mean arterial blood pressure (MAP), renal, mesenteric, and hindquarters Doppler shifts were measured by a transducer amplifier (13–4615–50; Gould, Cleveland, OH, USA), a Doppler flowmeter (Crystal Biotech, Holliston, MA, USA), and a VF‐1 mainframe (pulse repetition frequency 125 kHz) fitted with high‐velocity (HVPD‐20) modules. These measurements were recorded by customized computer software (IdeeQ; Maastricht Instruments, Maastricht, The Netherlands). Raw data were sampled by IdeeQ every 2 ms, averaged, and stored to disc every cardiac cycle. Changes in vascular conductance in the renal (RVC), mesenteric (MVC), and hindquarter (HVC) vascular beds, respectively, were calculated from the changes in MAP and Doppler shift.

### 2.4 Experimental protocol

Experiments were run in six studies, each lasting 3 days; within each study was a contemporaneous vehicle control (5% propylene glycol, 2% Tween 80 in sterile saline). Experiments were run with treatment groups of 8 to 10 rats.

#### 2.4.1 Study 1: The effect of β_1_ antagonist CGP 20712A (200 µg.kg^−1^ bolus; 100 µg.kg^−1^.h^−1^, 90 min infusion) on the hemodynamic profile of A_2A_ agonist CGS 21680

Eight animals were used to measure the cardiovascular responses to CGS 21680 in the presence or absence of CGP 20712A. Following a period of baseline recordings, rats were randomized into two groups. **Group 1** received vehicle intravenous bolus (0.1 ml provided over 5 s) followed by 90 min intravenous infusion (0.4 ml.h^−1^) on day 1 and CGP 20712A intravenous bolus (200 µg.kg^−1^) (0.1 ml provided over 5 s) followed by 90 min intravenous infusion (100 µg.kg^−1^.h^−1^) (0.4 ml.h^−1^) on day 3. **Group 2** received CGP 20712A (200 µg.kg^−1^, 0.1 ml i.v. bolus; 100 µg.kg^−1^.h^−1^, 0.4 ml.h^−1^ i.v. infusion) on day 1 and vehicle (0.1 ml i.v. bolus; 0.4 ml.h^−1^ i.v. infusion) on day 3. After 90 min all groups received intravenous infusions (0.1 ml.min^−1^) of CGS 21680 (0.1 (low), 0.3 (mid), and 1.0 (high) µg.kg^−1^ min^−1^). Each dose of CGS 21680 was given as a 3 min infusion. Cardiovascular recordings were continued for a further 4 h after administration of CGS 21680.

#### 2.4.2 Study 2: The effect of β_2_ antagonist ICI 118 551 (2.0 mg.kg^−1^ bolus; 1.0 mg.kg^−1^.h^−1^, 90 min infusion) on the hemodynamic profile of A_2A_ agonist CGS 21680

Nine animals were used to assess the cardiovascular responses to CGS 21680 in the presence or absence of ICI 118,551. After a period of baseline recordings, rats were randomized into two groups. **Group 1** received vehicle intravenous bolus (0.1 ml provided over 5 s) followed by 90 min intravenous infusion (0.4 ml.h^−1^) on day 1 and an ICI 118,551 intravenous bolus (2.0 mg.kg^−1^) (0.1 ml provided over 5 s) followed by 90 min intravenous infusion (1.0 mg.kg^−1^.h^−1^) (0.4 ml.h^−1^) on day 3. **Group 2** received ICI 118,551 (2.0 mg.kg^−1^, 0.1 ml i.v. bolus; 1 mg.kg^−1^.h^−1^, 0.4 ml.h^−1^ i.v. infusion) on day 1 and vehicle (0.1 ml i.v. bolus; 0.4 ml.h^−1^ i.v. infusion) on day 3. Approximately 90 min after the initial bolus of vehicle or ICI 118,551, all groups received intravenous infusions (0.1 ml.min^−1^) of CGS 21680 (0.1 (low), 0.3 (mid), and 1.0 (high) µg.kg^−1^ min^−1^). Each dose was infused for 3 min. Hemodynamic recordings were made for a further 4 h following the completion of the CGS 21680 intravenous infusion period.

#### 2.4.3 Study 3: The effect of β antagonist propranolol (1.0 mg.kg^−1^bolus; 0.5 mg.kg^−1^.h^−1^, 90 min infusion) on the hemodynamic profile of A_2A_ agonist CGS 21680

Ten animals were used to assess the cardiovascular responses to CGS 21680 in the presence or absence of propranolol. After a period of baseline recordings, rats were randomized into two groups. **Group 1** received vehicle intravenous bolus (0.1 ml provided over 5 s) followed by 90 min intravenous infusion (0.4 ml.h^−1^) on day 1 and a propranolol intravenous bolus (1.0 mg.kg^−1^) (0.1 ml provided over 5 s) followed by 90 min intravenous infusion (0.5 mg.kg^−1^.h^−1^) (0.4 ml.h^−1^) on day 3. **Group 2** received propranolol (1.0 mg.kg^−1^, 0.1 ml i.v. bolus; 0.5 mg.kg^−1^.h^−1^, 0.4 ml.h^−1^ i.v. infusion) on day 1 and vehicle (0.1 ml i.v. bolus; 0.4 ml.h^−1^ i.v. infusion) on day 3. Approximately 90 min after the initial bolus of vehicle or propranolol, all groups received intravenous infusions (0.1 ml.min^−1^) of CGS 21680 (0.1 (low), 0.3 (mid), and 1.0 (high) µg.kg^−1^ min^−1^). Each dose was infused for 3 min. Hemodynamic recordings were made for a further 4 h following the completion of the CGS 21680 intravenous infusion period.

#### 2.4.4 Study 4: The effect of β_1_ antagonist CGP 20712A (200 µg.kg^−1^ bolus; 100 µg.kg^−1^.h^−1^, 90 min infusion) on the hemodynamic profile of A_2B_ agonist BAY 60–6583

Eight animals were used to measure the cardiovascular responses to BAY 60–6583 in the presence or absence of CGP 20712A. Following a period of baseline, rats were randomized into two groups. **Group 1** received vehicle intravenous bolus (0.1 ml provided over 5 s) followed by 90 min intravenous infusion (0.4 ml.h^−1^) on day 1 and CGP 20712A intravenous bolus (200 µg.kg^−1^) (0.1 ml provided over 5 s) followed by 90 min intravenous infusion (100 µg.kg^−1^.h^−1^) (0.4 ml.h^−1^) on day 3. **Group 2** received CGP 20712A (200 µg.kg^−1^, 0.1 ml i.v. bolus; 100 µg.kg^−1^.h^−1^, 0.4 ml.h^−1^ i.v. infusion) on day 1 and vehicle (0.1 ml i.v. bolus; 0.4 ml.h^−1^ i.v. infusion) on day 3. After 90 min all groups received intravenous infusions (0.1 ml.min^−1^) of BAY 60–6583 (4.0 (low), 13.3 (mid), and 40.0 (high) µg.kg^−1^ min^−1^). Each dose of BAY 60–6583 was given as a 3 min infusion. Cardiovascular recordings were continued for a further 4 h after administration of BAY 60–6583.

#### 2.4.5 Study 5: The effect of β_2_ antagonist ICI 118,551 (2.0 mg.kg^−1^ bolus; 1.0 mg.kg^−1^.h^−1^, 90 min infusion) on the haemodynamic profile of A_2B_ agonist BAY 60–6583

Eight animals were used to assess the cardiovascular responses to BAY 60–6583 in the presence or absence of ICI 118,551. After a period of baseline recordings, rats were randomized into two groups. **Group 1** received vehicle intravenous bolus (0.1 ml provided over 5 s) followed by 90 min intravenous infusion (0.4 ml.h^−1^) on day 1 and an ICI 118,551 intravenous bolus (2.0 mg.kg^−1^) (0.1 ml provided over 5 s) followed by 90 min intravenous infusion (1.0 mg.kg^−1^.h^−1^) (0.4 ml.h^−1^) on day 3. **Group 2** received ICI 118,551 (2.0 mg.kg^−1^, 0.1 ml i.v. bolus; 1.0 mg.kg^−1^.h^−1^, 0.4 ml.h^−1^ i.v. infusion) on day 1 and vehicle (0.1 ml i.v. bolus; 0.4 ml.h^−1^ i.v. infusion) on day 3. Approximately 90 min after the initial bolus of vehicle or ICI 118,551, all groups received intravenous infusions (0.1 ml.min^−1^) of BAY 60–6583 (4.0 (low), 13.3 (mid), and 40.0 (high) µg.kg^−1^ min^−1^). Each dose was infused for 3 min. Hemodynamic recordings were made for a further 4 h following the completion of the BAY 60–6583 intravenous infusion period.

#### 2.4.6 Study 6: The effect of β antagonist propranolol (1.0 mg.kg^−1^ bolus; 0.5 mg.kg^−1^.h^−1^, 90 min infusion) on the hemodynamic profile of A_2B_ agonist BAY 60–6583

Ten animals were used to assess the cardiovascular responses to BAY 60–6583 in the presence or absence of propranolol. After a period of baseline recordings, rats were randomized into two groups. **Group 1** received vehicle intravenous bolus (0.1 ml provided over 5 s) followed by 90 min intravenous infusion (0.4 ml.h^−1^) on day 1 and a propranolol intravenous bolus (1 mg.kg^−1^) (0.1 ml provided over 5 s) followed by 90 min intravenous infusion (0.5 mg.kg^−1^.h^−1^) (0.4 ml.h^−1^) on day 3. **Group 2** received propranolol (1.0 mg.kg^−1^, 0.1 ml i.v. bolus; 0.5 mg.kg^−1^.h^−1^, 0.4 ml.h^−1^ i.v. infusion) on day 1 and vehicle (0.1 ml i.v. bolus; 0.4 ml.h^−1^ i.v. infusion) on day 3. Approximately 90 min after the initial bolus of vehicle or propranolol, all groups received intravenous infusions (0.1 ml.min^−1^) of BAY 60–6583 (4.0 (low), 13.3 (mid), and 40.0 (high) µg.kg^−1^ min^−1^). Each dose was infused for 3 min. Hemodynamic recordings were made for a further 4 h following the completion of the BAY 60–6583 intravenous infusion period.

### 2.5 NanoBRET ligand binding studies

The evaluation of the binding of β‐adrenoceptor ligands to rat Nanoluciferase‐tagged adenosine A_2A_ and A_2B_ receptors expressed in HEK293T cells was performed as previously described in Cooper et al. 2022.^10^ Briefly, competition binding experiments were performed with 50 nM CA200645, in the presence or absence of increasing concentrations of ICI 118,551, CGP 20712A or propranolol, in HEPES‐ buffered saline solution (HBSS; 147 mM NaCl, 5 mM KCl, 1.3 mM CaCl_2_, 1 mM MgSO_4_, 10 mM HEPES, 2 mM sodium pyruvate,1.43 mM NaHCO_3_, 10 mM d‐glucose, pH 7.45, supplemented with 0.1% BSA). Cells were incubated with ligand for 2 h at 37°C. Furimazine was then added to each well to give a final concentration of 10 μM. The cells were incubated for a further 5 min at 37°C. A PHERAstar FS plate reader (BMGLabtech) was used to measure the resulting bioluminescence resonance energy transfer (BRET) using filtered light emissions at 460 nm (80 nm bandpass) and >610 nm (longpass) at 37°C. The ratio between the >610 nm emission and the 460 nm emission provided the raw BRET data for each experiment.

### 2.6 Data analysis

All in vivo data were collected and analysed using IdeeQ software (Maastricht Instruments, Maastricht University, NL). For all experiments, time‐averaged data are shown as changes from baseline [HR (beats.min^−1^); MAP (mmHg); VC (%)]. Statistical comparisons between groups of animals were performed on the integrated changes over specified time periods. A Friedman’s nonparametric repeated‐measures analysis of variance was used for within‐group comparisons, and a Wilcoxon rank‐sum test for integrated area under or above curve analysis was used for comparisons between groups. A Wilcoxon test was also performed for comparisons between groups at specific time points. Vascular conductances were calculated from the MAP and Doppler shift (flow) data. A value of *p *< .05 was considered significant.

Key protein targets and ligands in this article are hyperlinked to corresponding entries in http://www.guidetopharmacology.org, the common portal for data from the IUPHAR/BPS Guide to PHARMACOLOGY^28^ and are permanently archived in the Concise Guide to PHARMACOLOGY 2019/20.^29^

## 3 RESULTS

**FIGURE 1:**
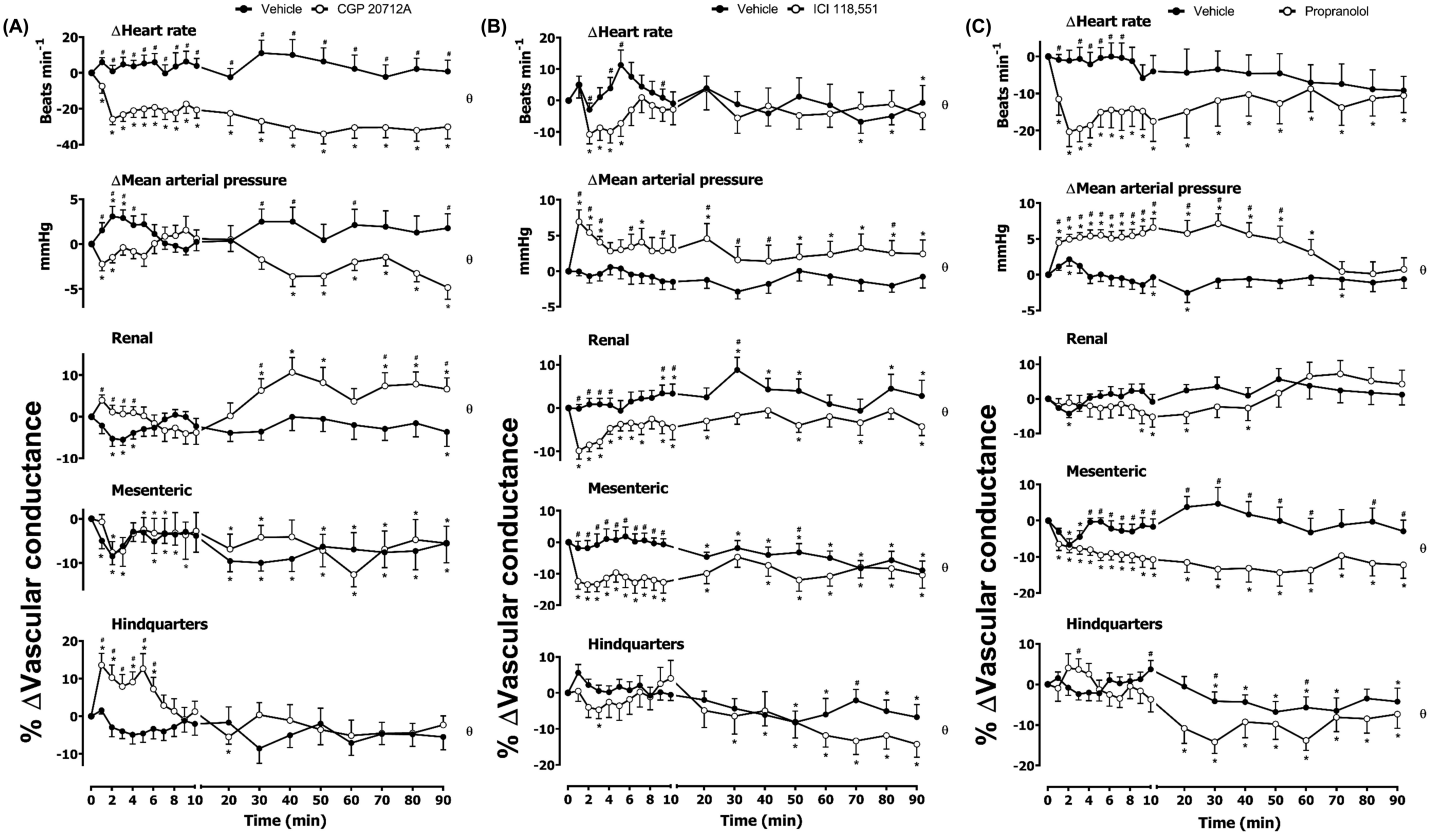
Cardiovascular responses to (A) CGP 20712A, (B) ICI 118,551, or (C) propranolol in conscious, freely moving rats. Rats were dosed with either (A) CGP 20712A (0.1 ml bolus dose of 200 μg.kg^−1^, followed by a 90 min infusion of 100 μg.kg^−1^.h^−1^, i.v., *n* = 16) or vehicle (0.1 ml bolus dose of 5% propylene glycol, 2% Tween 80 in sterile saline, *n* = 16); (B) ICI 118,551 (0.1 ml bolus dose of 2.0 mg.kg^−1^, followed by a 90 min infusion of 1.0 mg.kg^−1^.h^−1^ i.v., *n* = 17) or vehicle; or (C) propranolol (0.1 ml bolus dose of 1.0 mg.kg^−1^, followed by a 90 min infusion of 0.5 mg.kg^−1^.h^−1^ i.v., *n* = 20) or vehicle, as described in the methods. The time course shows the responses over the 90 min period during the infusion. Data points are mean and vertical bars represent SEM. *p < .05 versus baseline (Friedman’s test). A Wilcoxon signed‐rank test was conducted between treated and vehicle control groups for a comparison of area under/over the curve (θ *p *< .05) and to determine differences at each time point (# *p <* .05, Wilcoxon T‐test equivalent).

**FIGURE 2:**
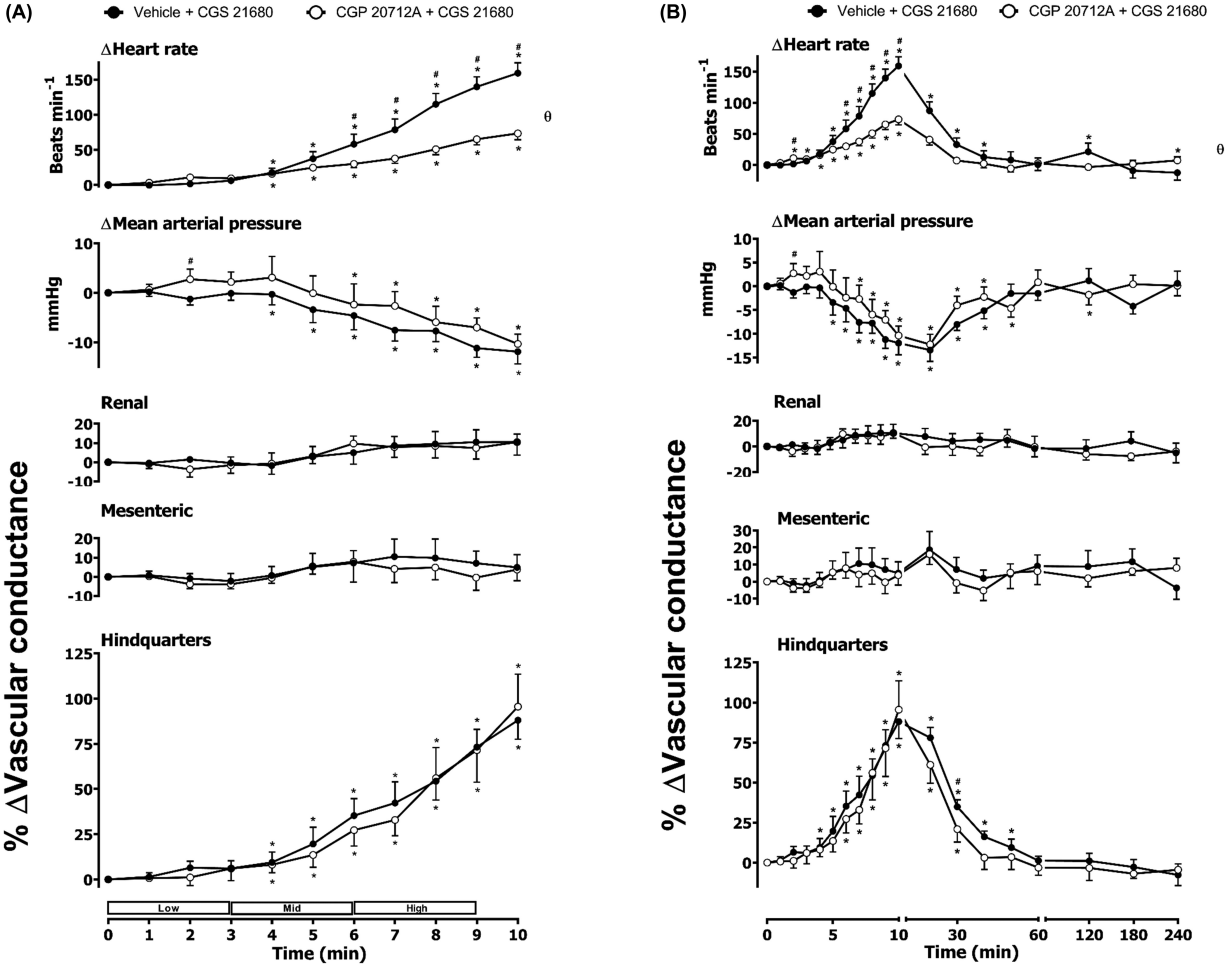
Cardiovascular responses to CGS 21680 in the presence or absence of CGP 20712A in conscious, freely moving rats. Rats were dosed with CGP 20712A (0.1 ml bolus dose of 200 μg.kg^−1^, followed by a 90 min infusion of 100 µg.kg^−1^.h^−1^ i.v., *n* = 8) or vehicle (0.1 ml bolus and a 90 min infusion of 5% propylene glycol, 2% Tween 80 in sterile saline, *n* = 8) as described in the methods. Following the 90 min infusion, all animals received an infusion of CGS 21680 (0.1, 0.3 and 1.0 μg.kg^−1^.min^−1^; each dose infused (i.v.) over 3 min. The time courses show (A) the treatment period and (B) the treatment period plus the extended 4 h recording period. Data points are mean and vertical bars represent SEM. *p < .05 versus baseline (Friedman’s test). A Wilcoxon signed‐rank test was conducted between treated and vehicle control groups for a comparison of area under/over the curve (θ *p *< .05) and to determine differences at each time point (# *p *< .05, Wilcoxon T‐test equivalent).

**FIGURE 3:**
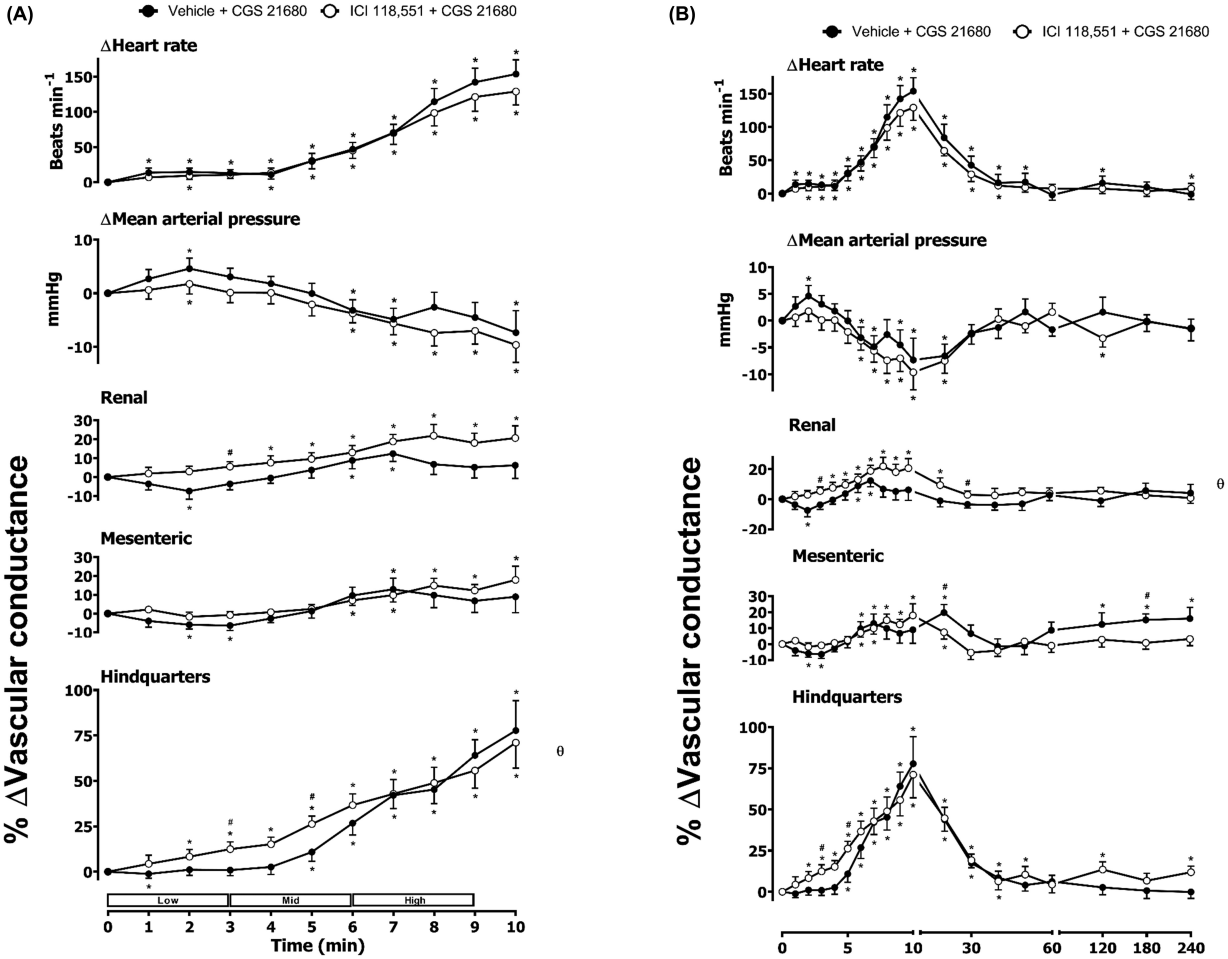
Cardiovascular responses to CGS 21680 in the presence or absence of ICI 118,551 in conscious, freely moving rats. Rats were dosed with ICI 118,551 (0.1 ml bolus dose of 2.0 mg.kg^−1^, followed by a 90 min infusion of 1.0 mg.kg^−1^.h^−1^ i.v., *n* = 9) or vehicle (0.1 ml bolus and a 90 min infusion of 5% propylene glycol, 2% Tween 80 in sterile saline, *n* = 9) as described in the methods. Following the 90 min infusion, all animals received an infusion of CGS 21680 (0.1, 0.3, and 1.0 μg.kg^−1^.min^−1^; each dose infused (i.v.) over 3 min. The time courses show (A) the treatment period and (B) the treatment period plus the extended 4 h recording period. Data points are mean and vertical bars represent SEM. *p < .05 versus baseline (Friedman’s test). A Wilcoxon signed‐rank test was conducted between treated and vehicle control groups for a comparison of area under/over the curve (θ *p *< .05) and to determine differences at each time point (# *p *< .05, Wilcoxon T‐test equivalent).

**FIGURE 4:**
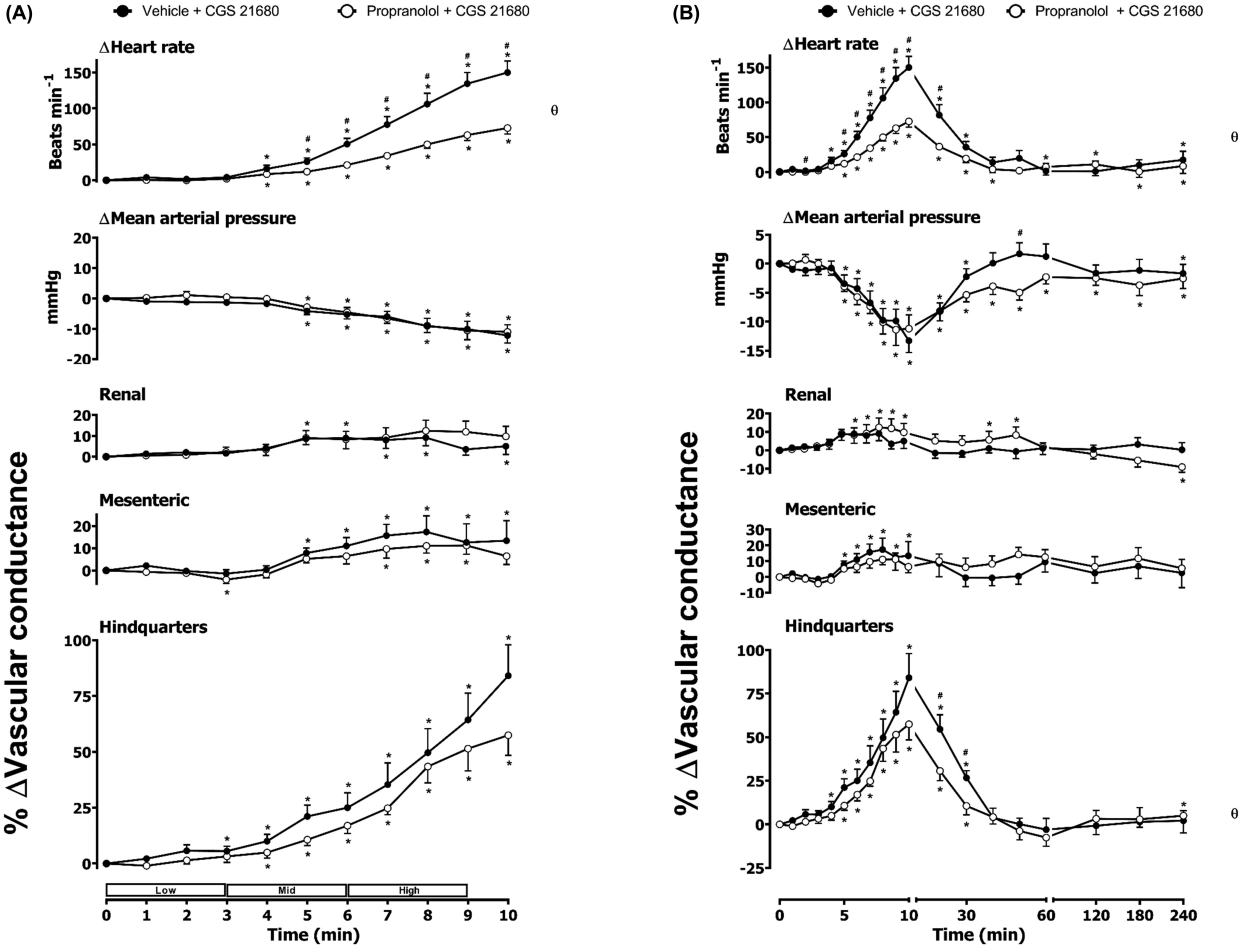
Cardiovascular responses to CGS 21680 in the presence or absence of propranolol in conscious, freely moving rats. Rats were dosed with propranolol (0.1 ml bolus dose of 1 mg.kg^−1^, followed by a 90 min infusion of 0.5 mg.kg^−1^.h^−1^ i.v., *n* = 10) or vehicle (0.1 ml bolus and a 90 min infusion of 5% propylene glycol, 2% Tween 80 in sterile saline, *n* = 10) as described in the methods. Following the 90 min infusion, all animals received an infusion of CGS 21680 (0.1, 0.3, and 1.0 μg.kg^−1^.min^−1^; each dose infused (i.v.) over 3 min. The time courses show (A) the treatment period and (B) the treatment period plus the extended 4 h recording period. Data points are mean and vertical bars represent SEM. *p < .05 versus baseline (Friedman’s test). A Wilcoxon signed‐rank test was conducted between treated and vehicle control groups for a comparison of area under/over the curve (θ *p <* .05) and to determine differences at each time point (# *p <* .05, Wilcoxon T‐test equivalent).

**FIGURE 5:**
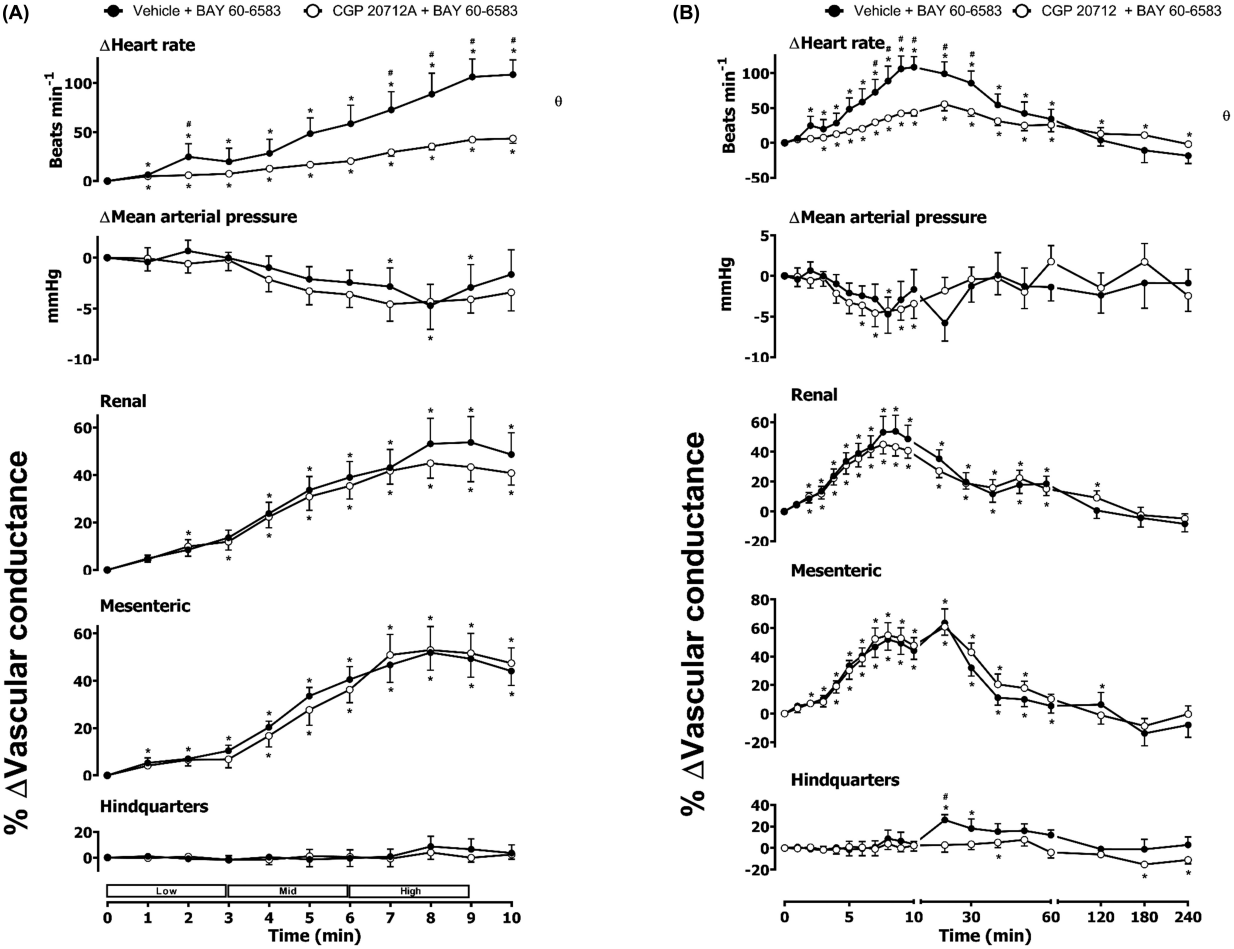
Cardiovascular responses to BAY 60–6583 in the presence or absence of CGP 20712A in conscious, freely moving rats. Rats were dosed with CGP 20712A (0.1 ml bolus dose of 200 μg.kg^−1^, followed by a 90 min infusion of 100 μg.kg^−1^.h^−1^ i.v., *n* = 8) or vehicle (0.1 ml bolus and a 90 min infusion of 5% propylene glycol, 2% Tween 80 in sterile saline, *n* = 8) as described in the methods. Following the 90 min infusion, all animals received an infusion of BAY 60–6583 (4.0, 13.3, and 40.0 μg.kg^−1^.min^−1^; each dose infused (i.v.) over 3 min. The time courses show (A) the treatment period and (B) the treatment period plus the extended 4 h recording period. Data points are mean and vertical bars represent SEM. *p < 0.05 versus baseline (Friedman’s test). A Wilcoxon signed‐rank test was conducted between treated and vehicle control groups for a comparison of area under/over the curve (θ *p <* .05) and to determine differences at each time point (# *p <* .05, Wilcoxon T‐test equivalent).

**FIGURE 6:**
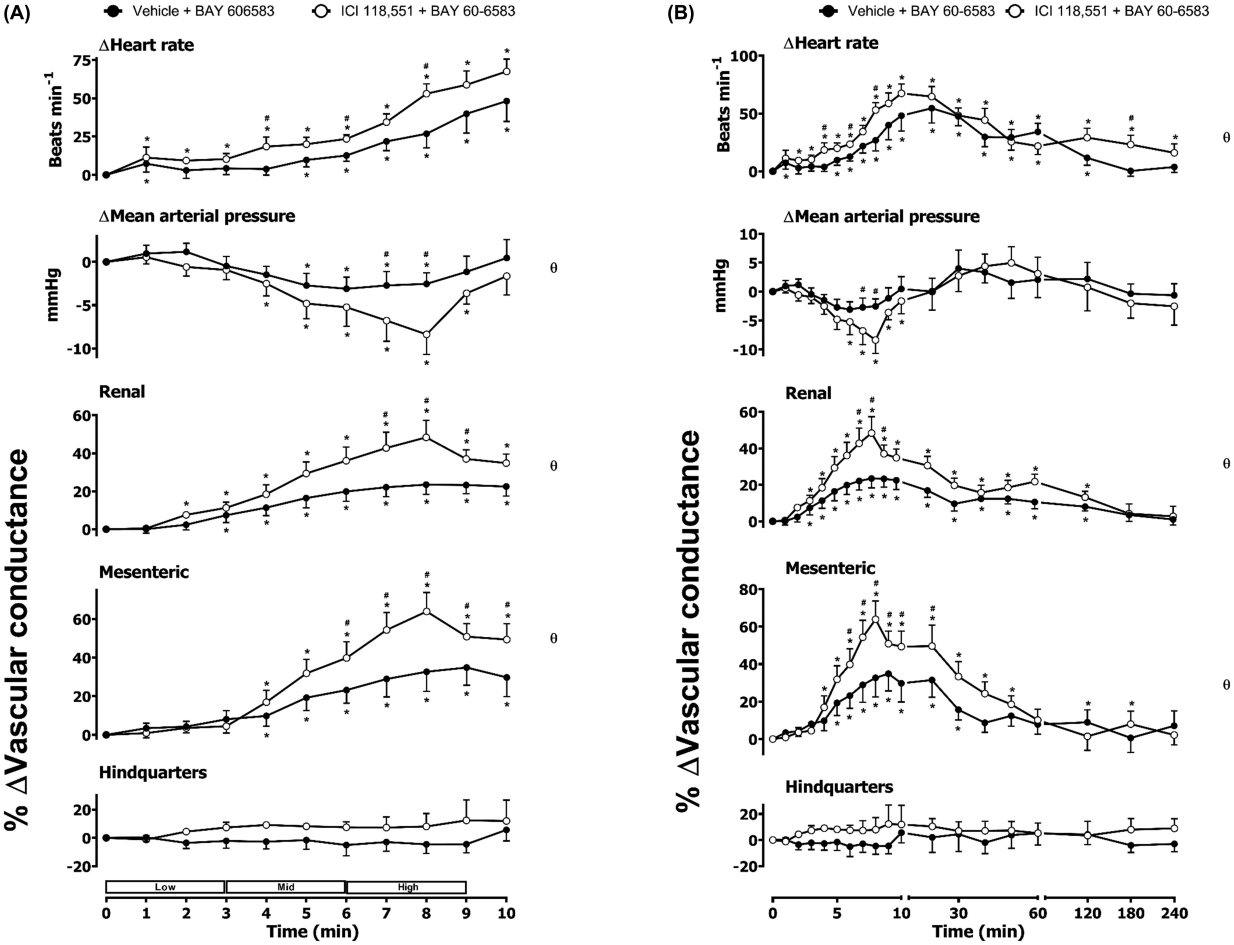
Cardiovascular responses to BAY 60–6583 in the presence or absence of ICI 118,551 in conscious, freely moving rats. Rats were dosed with ICI 118,551 (0.1 ml bolus dose of 2.0 mg.kg^−1^, followed by a 90 min infusion of 1.0 mg.kg^−1^.h^−1^ i.v., *n* = 8) or vehicle (0.1 ml bolus and a 90 min infusion of 5% propylene glycol, 2% Tween 80 in sterile saline, *n* = 8) as described in the methods. Following the 90 min infusion, all animals received an infusion of BAY 60–6583 (4.0, 13.3, and 40.0 μg.kg^−1^.min^−1^; each dose infused (i.v.) over 3 min. The time courses show (A) the treatment period and (B) the treatment period plus the extended 4 h recording period. Data points are mean and vertical bars represent SEM. *p < 0.05 versus baseline (Friedman’s test). A Wilcoxon signed‐rank test was conducted between treated and vehicle control groups for a comparison of area under/over the curve (θ *p <* .05) and to determine differences at each time point (# *p <* .05, Wilcoxon T‐test equivalent).

**FIGURE 7:**
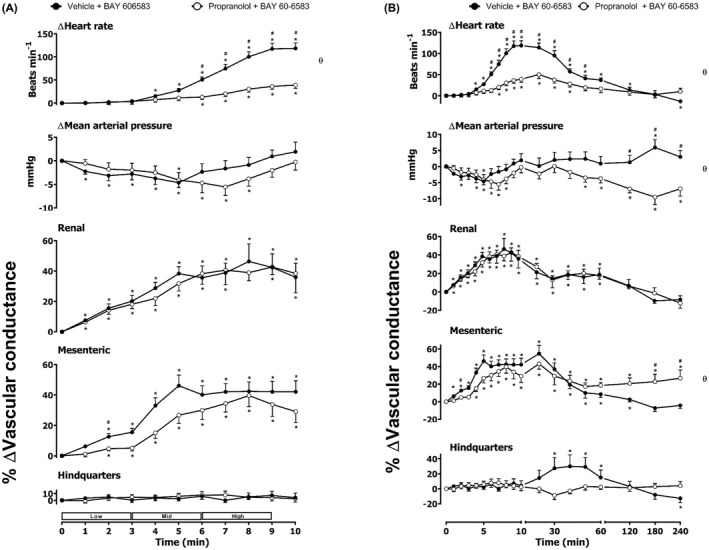
Cardiovascular responses to BAY 60–6583 in the presence or absence of propranolol in conscious, freely moving rats. Rats were dosed with propranolol (0.1 ml bolus dose of 1 mg.kg^−1^, followed by a 90 min infusion of 0.5 mg.kg^−1^.h^−1^ i.v., *n* = 10) or vehicle (0.1 ml bolus and a 90 min infusion of 5% propylene glycol, 2% Tween 80 in sterile saline, *n* = 10) as described in the methods. Following the 90 min infusion, all animals received an infusion of BAY 60–6583 (4.0, 13.3, and 40.0 μg.kg^−1^.min^−1^; each dose infused (i.v.) over 3 min. The time courses show (A) the treatment period and (B) the treatment period plus the extended 4 h recording period. Data points are mean and vertical bars represent SEM. *p < .05 versus baseline (Friedman’s test). A Wilcoxon signed‐rank test was conducted between treated and vehicle control groups for a comparison of area under/over the curve (θ *p <* .05) and to determine differences at each time point (# *p <* .05, Wilcoxon T‐test equivalent).

Treatment of rats with the selective β_1_‐adrenoceptor antagonist CGP 20712A confirmed that there was a significant basal sympathetic tone involving β_1_‐adrenoceptors in maintaining HR, and showed modest increases in renal and hindquarters vascular conductance (Figure 1). In contrast, the selective β_2_‐adrenoceptor antagonist ICI 118,551 produced a short‐lived transient decrease in HR and small decreases in vascular conductance in all three vascular beds (Figure 1). The data obtained with propranolol were consistent with antagonism of β_1_‐adrenoceptors in the heart and β_2_‐adrenoceptor antagonism in each vascular bed (Figure 1). Baseline cardiovascular variables for the combined results of studies before the administration of CGP 20712A, ICI 118,551, or propranolol are summarized in Table 1 and correspond to the baseline values shown in Figure 1. Baseline cardiovascular variables before the administration of CGP 20712A, ICI 118,551 or propranolol, adenosine receptor agonists, or their corresponding vehicle controls are shown in Table 2; these baseline values correspond to the baseline values shown in Figures 2, 3, 4, 5, 6, 7.

**TABLE 1:** Cardiovascular variables prior to administration of β‐antagonists for combined study datasets

Baseline (t =0)	Combination of Studies 1 & 4				Combination of Studies 2 & 5				Combination of Studies 3 & 6			
	Vehicle		CGP 20712A		Vehicle		ICI 118,551		Vehicle		Propranolol	
	Mean ± SEM	*n*	Mean ± SEM	*n*	Mean ± SEM	*n*	Mean ± SEM	*n*	Mean ± SEM	*n*	Mean ± SEM	*n*
HR (beats min^−1^)	333 ± 5	16	338 ± 8	16	330 ± 6	17	332 ± 7	17	348 ± 5	20	344 ± 5	20
MAP (mmHg)	102 ± 2	16	104 ± 2	16	104 ± 3	17	103 ± 2	17	104 ± 1	20	103 ± 2	20
RVC (U)	81 ± 7	15	73 ± 5	15	93 ± 8	16	89 ± 7	16	83 ± 7	16	85 ± 5	16
MVC (U)	82 ± 7	14	75 ± 8	14	87 ± 7	17	83 ± 5	17	93 ± 7	18	102 ± 7	18
HVC (U)	44 ± 5	14	42 ± 4	14	44 ± 4	16	43 ± 4	16	54 ± 5	19	54 ± 5	19

Note

Values are mean ± SEM. Units of vascular conductance (VC) are kHz. mmHg^−1^ × 10^3^. *n* = 14–20 per combined group.

Wilcoxson matched‐pairs signed‐rank test. *p < .05 versus corresponding vehicle group.

Abbreviations: HR, heart rate; HVC, hindquarters vascular conductance; MAP, mean arterial pressure; MVC, mesenteric vascular conductance; RVC, renal vascular conductance; U, units; VC, vascular conductance.

**TABLE 2:** Cardiovascular variables prior to administration of β‐antagonists (top). Cardiovascular variables prior to infusion of A_2A_ agonist CGS 21680 (Study 1, 2, 3) or A_2B_ agonist BAY 60–6583 (Study 4, 5, 6) (bottom)

Baseline (t = 0)	Study 1				Study 2				Study 3				Study 4				Study 5				Study 6			
	Vehicle		CGP 20712A		Vehicle		ICI 118,551		Vehicle		Propranolol		Vehicle		CGP 20712A		Vehicle		ICI 118,551		Vehicle		Propranolol	
	Mean ± SEM	*n*	Mean ± SEM	*n*	Mean ± SEM	*n*	Mean ± SEM	*n*	Mean ± SEM	*n*	Mean ± SEM	*n*	Mean ± SEM	*n*	Mean ± SEM	*n*	Mean ± SEM	*n*	Mean ± SEM	*n*	Mean ± SEM	*n*	Mean ± SEM	*n*
HR (beats min^−1^)	337 ± 8	8	328 ± 11	8	320 ± 5	9	320 ± 8	9	350 ± 7	10	344 ± 7	10	329 ± 7	8	348 ± 12	8	341 ± 12	8	346 ± 9	8	346 ± 6	10	343 ± 7	10
MAP (mmHg)	101 ± 2	8	107 ± 3	8	101 ± 3	9	97 ± 2	9	105 ± 3	10	104 ± 3	10	102 ± 3	8	102 ± 1	8	109 ± 5	8	110 ± 3	8	103 ± 1	10	102 ± 3	10
RVC (U)	83 ± 10	7	69 ± 8*	7	88 ± 9	9	92 ± 8	9	85 ± 7	10	86 ± 7	10	79 ± 9	8	78 ± 6	8	100 ± 16	7	86 ± 12	7	83 ± 12	6	84 ± 7	6
MVC (U)	84 ± 7	7	73 ± 7	7	97 ± 10	9	89 ± 8	9	80 ± 7	9	103 ± 8	9	80 ± 13	7	78 ± 15	7	76 ± 8	8	76 ± 6	8	106 ± 11	9	102 ± 12	9
HVC (U)	36 ± 7	7	32 ± 5	7	48 ± 3	9	48 ± 4	9	51 ± 5	10	53 ± 7	10	53 ± 6	7	52 ± 5	7	39 ± 7	7	37 ± 7	7	55 ± 8	9	55 ± 8	9
HR (beats min^−1^)	329 ± 9	8	303 ± 9*	8	312 ± 5	9	316 ± 8	9	341 ± 7	10	333 ± 8	10	331 ± 10	8	309 ± 8	8	340 ± 11	8	334 ± 11	8	336 ± 8	10	332 ± 8	10
MAP (mmHg)	103 ± 1	8	99 ± 3	8	99 ± 2	9	100 ± 3	9	104 ± 2	10	101 ± 3	10	103 ± 2	8	99 ± 2	8	107 ± 5	8	110 ± 2	8	100 ± 2	10	107 ± 3*	10
RVC (U)	82 ± 10	7	81 ± 11	7	88 ± 8	9	87 ± 8	9	85 ± 7	10	89 ± 8	10	76 ± 8	8	79 ± 6	8	103 ± 16	7	83 ± 12*	7	81 ± 12	6	85 ± 7	6
MVC (U)	78 ± 4	7	71 ± 6	7	86 ± 9	9	82 ± 9	9	81 ± 8	9	99 ± 11	9	74 ± 11	7	71 ± 13	7	71 ± 6	8	67 ± 8	8	101 ± 12	9	82 ± 11	9
HVC (U)	34 ± 6	7	33 ± 6	7	47 ± 3	9	43 ± 5	9	51 ± 5	10	47 ± 4	10	49 ± 5	7	49 ± 4	7	35 ± 7	7	31 ± 6	7	54 ± 9	9	47 ± 7	9

Note

Values are mean ± SEM. Units of vascular conductance (VC) are kHz. mmHg^−1^ × 10^3^. *n* = 6–10 per group.

Wilcoxson matched‐pairs signed‐rank test. *p < .05 versus corresponding vehicle group.

Abbreviations: HR, heart rate; HVC, hindquarters vascular conductance; MAP, mean arterial pressure; MVC, mesenteric vascular conductance; RVC, renal vascular conductance; U, units.

^†^ In some instances agonist administration was delayed past 90 min in the case of movement to allow the rat to settle.

### 3.1 Effect of β‐adrenoceptor antagonists on the hemodynamic profile of the A_2A_ agonist CGS 21680

Consistent with our previous observations,^10^ the selective A_2A_ agonist CGS 21680 produced a marked increase in vascular conductance in the hindquarters of conscious, freely moving rats without a significant effect on blood flow in the renal and mesenteric circulations (Figure 2). This change in hindquarters VC was accompanied by a significant increase in HR and a fall in MAP. The addition of the β_1_ adrenoceptor‐selective antagonist CGP 20712A, 90 min prior to the increasing concentrations of the A_2A_ agonist CGS 21680, significantly attenuated the A_2A_ agonist‐induced increase in HR (*p *< .05; Figure 2). However, no significant difference was observed in MAP, RVC, MVC, and HVC between vehicle‐pretreated rats and CGP 20712A‐pretreated rats (Figure 2). This dose of CGP 20712A was selected as it has previously been shown to produce a highly selective antagonism of cardiac β_1_‐adrenoceptors in vivo in the rat.^30^, ^31^ Selective antagonism of β_2_‐adrenoceptors with ICI 118,551,^30^, ^31^ however, produced a small but significant increase in the RVC response mediated by CGS 21680, but did not alter HVC, HR, or MAP (Figure 3). Pretreatment with the non‐selective β‐blocker propranolol produced a marked (*p *< .05) decrease in HR with no significant change in MAP (Figure 4). Propranolol did, however, produce a small reduction (*p *< .05) in the hindquarters vasodilatation (Figure 4).

### 3.2 **Effect of**β**‐adrenoceptor antagonists on the hemodynamic profile of the A_2B_ agonist BAY 60–6583**

As noted previously,^10^ the A_2B_ agonist BAY 60–6583 produced a large increase in HR as well as significant increases in RVC and MVC (Figure 5). There was no significant effect of BAY 60–6583 on MAP or hindquarters blood flow (Figure 5). Pre‐treatment with the β_1_‐adrenoceptor‐selective antagonist CGP 20712A produced a significant attenuation (*p *< .05) of the heart rate response to BAY 60–6583 without any change in the vasodilatation induced in the renal and mesenteric circulations (Figure 5). The β_2_‐adrenoceptor selective antagonist ICI 118,551 significantly increased the HR, RVC, and MVC responses induced by the A_2B_ agonist (Figure 6). Antagonism of both β_1_‐ and β_2_‐adrenoceptors produced a marked decrease in the HR response to BAY 60–6583 and a small reduction in MAP in the presence of both propranolol and BAY 60–6583 (Figure 7). Propranolol did not, however, attenuate the vasodilatation induced by the selective A_2B_‐receptor agonist in the renal and mesenteric circulations.

### 3.3 NanoBRET ligand binding studies

To verify that the β‐adrenoceptor ligands do not directly bind to either of the two A_2_ adenosine receptors, we also performed NanoBRET ligand binding studies utilizing rat N‐terminal Nanoluciferase‐tagged A_2A_ and A_2B_ receptors, as described previously.^10^ CGP 20712A, ICI 118,551, and propranolol had no effect on the specific binding of the non‐selective fluorescent ligand CA200645 (50 nM) to either rat receptor expressed in HEK293T cells (*n *= 5) at concentrations up to 100 μM, suggesting that none of the β‐adrenoceptor ligands tested directly bind to either of the two rat A_2_ receptors.

## 4 DISCUSSION

**FIGURE 8:**
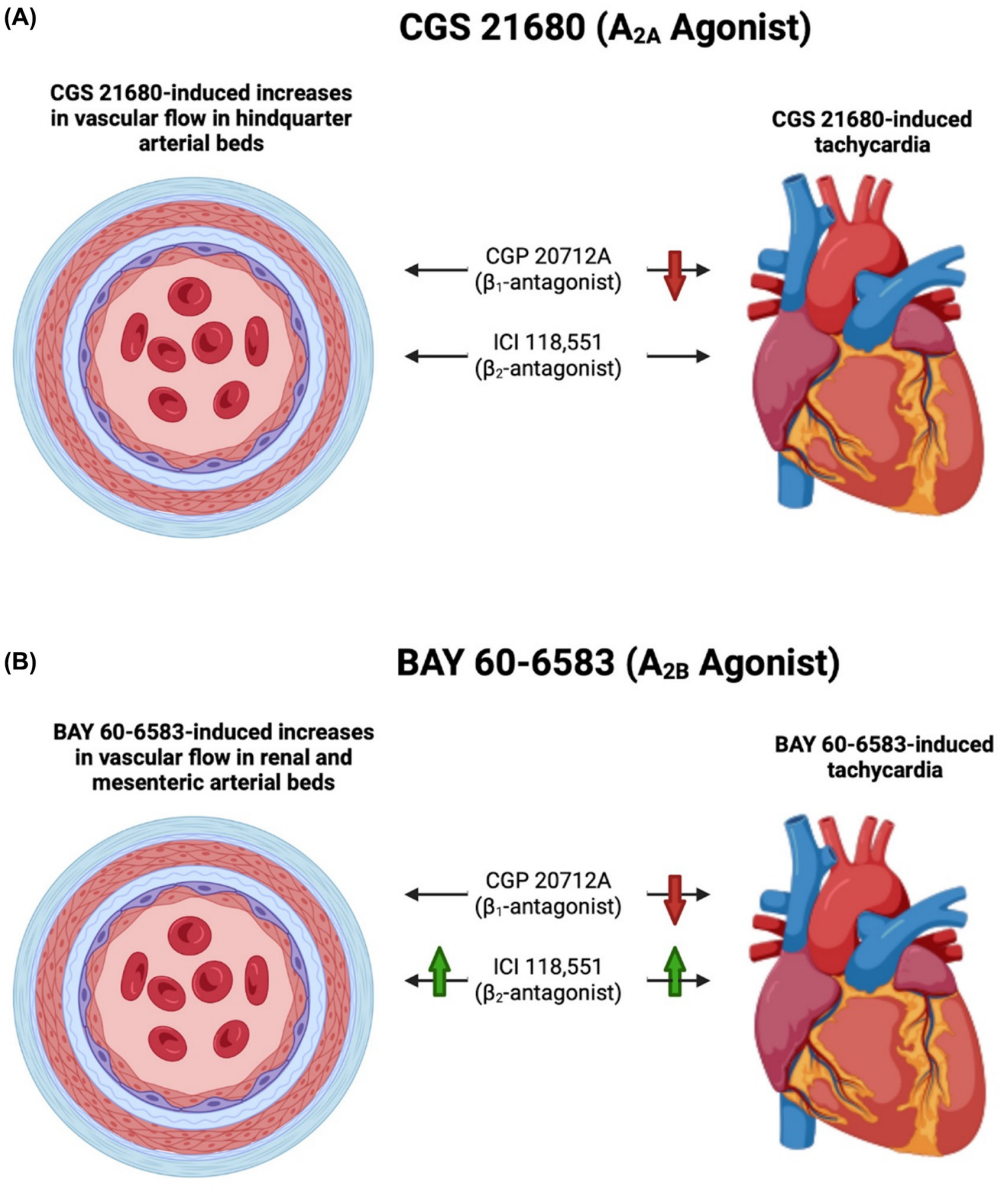
Summary of the key experimental findings. The impact of β_1_‐antagonist, CGP 20712A and β_2_‐antagonist, ICI 118,551 on the heart rate and vascular conductance profiles of (A) A_2A_ agonist, CGS 21680, and (B) A_2B_ agonist, BAY 60–6583 (created with Biorender.com).

Consistent with our previous observations,^10^ the selective A_2A_ agonist CGS 21680 produced a marked increase in HR that was associated with a fall in MAP in conscious rats. A similar effect has been reported with the A_2A_ receptor agonist regadenoson.^32^ These authors went on to suggest that the increase in HR was mediated by a direct A_2A_‐receptor‐mediated stimulation of the sympathetic nervous system since the HR response, but not the fall in MAP, was attenuated by the β‐blocker metoprolol.^32^ This result suggests that a baroreflex‐mediated increase in HR is not the sole reason for the tachycardia observed with CGS 21680.^32^ In the present study, we therefore investigated the effect of selective β_1_‐ and β_2_‐ selective antagonists on the different cardiovascular responses to the A_2A_‐selective agonist CGS 21680 and the A_2B_‐selective agonist BAY 60–6583 to determine the extent to which they are secondary to sympathetic excitation (Figure 8).

To do this, we chose a dosage regimen with CGP 20712A and ICI 118,551 that produced highly selective antagonism in vivo of β_1_‐ and β_2_‐ adrenoceptor cardiovascular responses respectively.^30^, ^31^ The selective β_1_‐adrenoceptor antagonist CGP 20712A significantly attenuated the HR response to CGS 21680 without changing the fall MAP, consistent with previous findings obtained using regadenoson.^32^ These data suggest that a direct activation of the sympathetic nervous system is indeed induced by A_2A_‐receptor stimulation. It was therefore important to determine what effect CGP 20712A or ICI 118,551 treatment would have on the regionally selective increase in vascular conductance in the hindquarters observed with CGS 21680 in conscious, freely moving rats. However, neither CGP 20712A nor ICI 118,551 had any effect on HVC. Furthermore, ICI 118,551 did not change the increase in HR or the fall in MAP induced by this A_2A_‐selective agonist. As expected, the non‐selective β‐blocker propranolol reduced the tachycardia induced by CGS 21680 without affecting MAP responses. However, propranolol did produce a small attenuation of the hindquarters vasodilatation, which may be related to its off‐target actions on other receptors, such as the 5‐HT_1B_ receptor.^33^

In the case of the A_2B_‐receptor selective agonist BAY 60–6583, the profile of cardiovascular responses was quite different to that obtained with CGS 21680.^10^ Although BAY 60–6583 produced a large increase in heart rate, this was not accompanied by a change in MAP consistent with the tachycardia being due to either a direct effect of A_2B_‐receptor activation in the heart or due to a similar excitation of the sympathetic nervous system described above for A_2A_‐receptors. In keeping with the latter hypothesis, CGP 20712A significantly attenuated the HR response to BAY 60–6583. In contrast with A_2A_‐receptor stimulation, A_2B_‐receptor activation produced increases in vascular conductance in the renal and mesenteric vascular beds without a change in the hindquarters. These changes in vascular conductance were increased by treatment with ICI 118,551, but were not altered by CGP 20712A. ICI 118,551 treatment also produced a small significant increase in the HR response to BAY 60–6583. It was notable that in the absence of adenosine receptor agonist treatment, ICI 118,551 produced a significant transient fall in HR and more prolonged decreased in both RVC and MVC. This is consistent with a significant β_2_‐adrenoceptor‐mediated sympathetic tone under basal conditions that is removed by ICI 118,551. It is therefore likely that the enhanced effects of BAY 60–6583 on HR, RVC, and MVC in the presence of ICI 118,551 is a consequence of the direct A_2B_‐receptor responses being able to achieve maximum responses from a lower baseline signal. This is also seen to some extent with CGS 21680 on the RVC where, in the presence of ICI 118,551 a small significant vasodilation is revealed in the presence of the β_2_‐adrenoceptor antagonist.

Taken together, these data suggest that the regionally selective effects of A_2A_ and A_2B_ receptor activation on vascular conductance are the result of direct activation of receptors in the respective vascular beds and are not secondary to activation of the sympathetic nervous system. In contrast, the tachycardia induced by both receptor subtypes seems to involve a component of direct sympathetic activation leading to stimulation of β_1_‐adrenoceptors in the heart. Adenosine has been shown to activate afferent nerve terminals in the kidney and heart leading to sympathetic activation,^34^, ^35^ but the exact mechanisms involved remains to be established. However, in the presence of a high dose of a selective β_1_‐adrenoceptor antagonist, which produces a marked and selective antagonism in β_1_‐adrenoceptor responses to isoprenaline in the heart,^30^ both A_2A_ and A_2B_ agonists still caused a small increase in HR, indicative of some direct chronotropic effect at the level of the heart.

Both A_2A_ and A_2B_ receptors are expressed in the heart and coronary arteries.^36^, ^37^ Indeed, the A_2A_ selective agonist regadenoson has been developed clinically as a coronary vasodilator for myocardial perfusion imaging.^38^ In atrial samples taken from patients with atrial fibrillation, an increase in expression of A_2A_ receptors has been reported, which is linked to abnormal calcium release from the sarcoplasmic reticulum.^39^, ^40^ A reduction in the expression of ventricular A_2A_‐receptors has also been observed in patients with chronic heart failure.^41^ Evidence for a direct role of A_2A_ receptors on cardiac contractility, however, remains unclear with contradictory reports of the effect of CGS 21680 on contractile activity and cyclic AMP accumulation in rat ventricular myocytes and isolated rat heart preparations.^42^, ^43^, ^44^, ^45^

BAY 60–6583 has previously been shown to be cardioprotective and to reduce reperfusion injury and myocardial infarct size in isolated rat hearts.^46^, ^47^, ^48^ A_2B_ receptor stimulation also inhibits proliferation and collagen synthesis in isolated cardiac fibroblasts.^49^, ^50^, ^51^ Furthermore, treatment with the A_2B_‐receptor antagonist GS‐6201 has been shown to improve cardiac function by preventing the remodeling and fibrogenesis that occurs following A_2B_ receptor activation.^52^ Interestingly, tissue‐specific knockout of A_2B_ receptors from both cardiomyocytes and vascular endothelial cells showed that A_2B_‐receptors were critical for ischemia‐reperfusion injury‐elicited cardioprotection, but reperfusion injury was also increased if A_2B_‐receptor signaling was knocked out from inflammatory cells.^53^ Selective activation of A_2B_‐receptors has also been demonstrated to play an important role in both renal and mesenteric ischemic reperfusion injury by improving capillary flow and function.^54^, ^55^ The fact that BAY 60–6583 can increase blood flow in both the renal and mesenteric circulation without producing a change in MAP^10^ suggests that use of selective A_2B_‐receptor agonists may be a promising approach for the treatment of acute kidney injury and mesenteric ischemia. Although A_2B_‐receptor agonism resulted in attenuation of ischemic kidney injury in a murine model^54^ and elicited renal vasodilation in conscious rats,^10^ the molecular mechanisms underlying A_2B_ receptor‐mediated renovascular protection are yet to be fully defined. As a result, addressing the specific role of A_2B_ signaling in renal ischemia‐reperfusion injury might be crucial to define the potential therapeutic uses of A_2B_ receptor agonists in the context of renal injury.

In summary, the present study has confirmed that the tachycardia induced by the selective A_2A_‐receptor agonist CGS 21680 is partly due to an activation of the sympathetic nervous system, and can be readily attenuated by the selective β_1_‐adrenoceptor antagonist CGP 20712A. In contrast, neither β_1_‐ nor β_2_‐selective antagonists had any significant effect on the fall in MAP or the increase in vascular conductance in the hindquarters vascular bed induced by CGS 21680. Similarly, the regionally selective vasodilator effects of the A_2B_‐selective agonist BAY 60–6583 in the renal and mesenteric circulations were not secondary to indirect sympathetic activation of β_1_‐ or β_2_‐adrenoceptors. Importantly, we have also shown that the large increase in HR produced by BAY 60–6583 can be markedly attenuated by selective β_1_‐adrenoceptor antagonists without significant effect on the changes in vascular conductance. Furthermore, infusion of BAY 60–6583 did not alter mean arterial pressure (in the presence or absence of β_1_‐adrenoceptor blockade) (Figure 8). These data suggest that the conjoint use of an A_2B_‐selective agonist and a β‐blocker might be an effective way to achieve a beneficial effect in the treatment of acute kidney injury or mesenteric ischemia.

### 4.1 Study limitations

Although in this rodent model our findings indicate that selective activation of A_2A_ and A_2B_‐receptors produces regionally selective vasodilatations and that specific A_2B_‐receptor vascular responses can be enhanced by β_2_‐adrenoceptor antagonism (Figure 8), it is important to note that ligand affinity and potency at adenosine receptors and other GPCRs can differ significantly between species and must be taken into account when considering the implications of this research.^56^, ^57^, ^58^ Furthermore, the acute studies presented here only give an indication of the short‐term impact of the ligands investigated. All four adenosine receptor subtypes are known to undergo agonist‐induced desensitization, internalization, and cellular trafficking, the effect of which may not be seen in these short‐term in vivo studies.^59^, ^60^ Therefore, future studies should include longer term dosing regimens and observations, in addition to clinical studies, to confirm that the conclusions presented here translate to the human condition.

## AUTHOR CONTRIBUTIONS

*Participated in research design*: SLC, JW, SJH, ESW, *Conducted experiments*: ESW, PP, SLC, *Performed data analysis*: ESW, PP, SLC, JW, *Wrote or contributed to the writing of the manuscript*: SJH, SLC, PP, JW, ESW.

## DISCLOSURES

The authors declare no conflicts of interest.

## ETHICS STATEMENT

All procedures were performed with approval of the University of Nottingham Animal Welfare and Ethical Review Board and performed in line with the Animals (Scientific Procedures) Act (1986), under UK Home Office approved Project License and Personal License authority. 53 rats were used during this study, and all animal experiments are reported in compliance with the ARRIVE guidelines^25^ and the editorial on reporting animal studies.^26^

## Data Availability

The data that support the findings of this study are available from the corresponding author upon reasonable request.
